# The single-strand DNA-binding protein SSB1 is involved in the expression of salivary gland radiation injury repair

**DOI:** 10.3389/fphar.2024.1471996

**Published:** 2024-10-15

**Authors:** Xian Wang, Yuetong Wang, Xianglin Zeng, Haoyu Lu, Dongqin Mo, Yuetao Li, Zhiqing Liu, Yude Huang, Kun Yu, Daiyou Wang

**Affiliations:** ^1^ College & Hospital of Stomatology, Guangxi Medical University, Nanning, Guangxi, China; ^2^ Guangxi Key Laboratory of Oral and Maxillofacial Rehabilitation and Reconstruction, Nanning, Guangxi, China; ^3^ Affiliated Stomatology Hospital of Guilin Medical University, Guilin, Guangxi, China

**Keywords:** single-strand DNA-binding protein 1, salivary gland, radioactive damage, DSB repair, γ-H2AX, PARP1

## Abstract

**Objectives:**

Single-strand DNA-binding protein 1 (SSB1) plays a crucial role in the cellular response to DNA damage. This study aimed to explore the expression and regulation of SSB1 in normal rat salivary gland tissues and tissues following radiation, with a specific emphasis on its involvement in the repair of salivary gland injury.

**Methods:**

A total of 45 adult SD rats were randomly assigned to one control group or eight experimental groups. In the control group, five rats were euthanized without irradiation, and their parotid gland tissues were collected for analysis. The experimental groups received a dose of 6 Gy of radiation targeting the head and neck region; subsequently, five rats from each group were euthanized hly to collect parotid gland tissue samples, resulting in a total of eight experimental groups. The expression levels of SSB1, γ-H2AX, and PARP1 in the parotid gland tissues were assessed via immunohistochemistry, while changes in SSB1 gene expression were quantified via RT-qPCR.

**Results:**

No significant morphological differences were observed between the two groups following HE staining. In the immunohistochemistry (IHC) analysis, notable tissue-specific variation in SSB1 expression was identified, with higher levels detected in the ducts than in the acini and connective tissue. The expression of SSB1 gene initially increased post-radiation before subsequently decreasing, ultimately returning to baseline levels, as corroborated by the RT-qPCR results. In contrast, γ-H2AX and PARP1 exhibited minimal expression in the control group; however, their expression peaked at 1 h in the experimental group before gradually declining to levels comparable to those of the control group.

**Conclusion:**

Radiation induces time-dependent upregulation of SSB1 expression in rat salivary glands, indicating that SSB1 may play a role in radiation-induced repair processes.

## 1 Introduction

Head and neck cancer (HNC) ranks as the sixth most prevalent form of cancer globally, with a disproportionately higher incidence rate observed in developing nations (Siegel et al., 2024). This cancer is commonly linked to elevated levels of tobacco and alcohol consumption (Jerjes et al., 2012). Radiotherapy is the primary or adjunctive treatment modality for patients with head and neck cancer. However, irradiation of the head and neck region often unavoidably affects normal tissue structures, leading to a range of radiation-induced injuries. Among these, acute and chronic dysfunctions of the salivary glands are relatively prevalent, presenting clinically as xerostomia, rampant caries, oral mucositis, dysphagia, altered taste perception, heightened susceptibility to oral infections, and malnutrition. These conditions significantly impact patients' quality of life (Vissink et al., 2010). While precise individualized radiotherapy has reached a consensus in recent years, and techniques such as intensity-modulated radiotherapy (IMRT) have been employed to spare normal tissues, such as salivary glands, and mitigate secondary side effects, clinical practice continues to involve radiation-induced salivary gland injuries.

In recent years, several studies have focused on the mechanism of radiation-induced salivary gland injury. Among these, the prevailing perspective revolves around DNA double-strand breaks. DNA, which serves as a repository of genetic information, is highly important for organism survival and reproduction. The study of DNA damage and its repair mechanisms is crucial for understanding how to maintain genome stability and prevent diseases such as cancer (Petermann et al., 2022). Ionizing radiation can elicit a cascade of cellular-level responses, with cellular DNA double-strand breaks (DSBs) being the predominant and most severe form of DNA damage (Ciccia and Elledge, 2010). The resistance of a cell to radiation is not only influenced by the DNA damage repair mechanism but also directly impacted by the ability of the cell to repair DSBs, which in turn affects its survival rate after radiation. Therefore, a comprehensive understanding of early events in DNA repair in normal tissues is essential for guiding tumor treatment methods and minimizing incidental damage to normal tissues during therapeutic radiotherapy.

Single-stranded DNA binding protein 1 (SSB1) is a member of the single-stranded DNA binding protein (SSB) family, with its encoding gene NABP2 located at chromosome 12q13.3 and spanning a length of 764 base pairs. This protein comprises two primary functional domains: the OB domain, which serves as the DNA-binding domain, and the more exposed hydroxyl-terminal domain, which facilitates protein-protein interactions (Ren et al., 2014). SSB1 plays a pivotal role in the DNA damage response. As an early responder, it promptly associates with exposed single-strand DNA to shield it from degradation by various nucleases present in the cellular milieu. Furthermore, it facilitates the recruitment of other repair proteins to the sites of DNA breaks, thereby enhancing the functionality of the repair mechanism at these damaged loci (Richard et al., 2011a; Richard et al., 2011b). In 2008, researchers initially identified SSB1 in higher eukaryotes. It is widely distributed across various cell types and tissues without confinement to specific cell types, indicating its importance in maintaining genomic stability to a certain extent (Richard et al., 2008). Moreover, the high conservation of SSB1 across diverse organisms suggests that its role in the DNA damage response and associated processes is essential and has been evolutionarily conserved, suggesting the existence of similar DNA damage response and repair mechanisms in various organisms.

**FIGURE 1:**
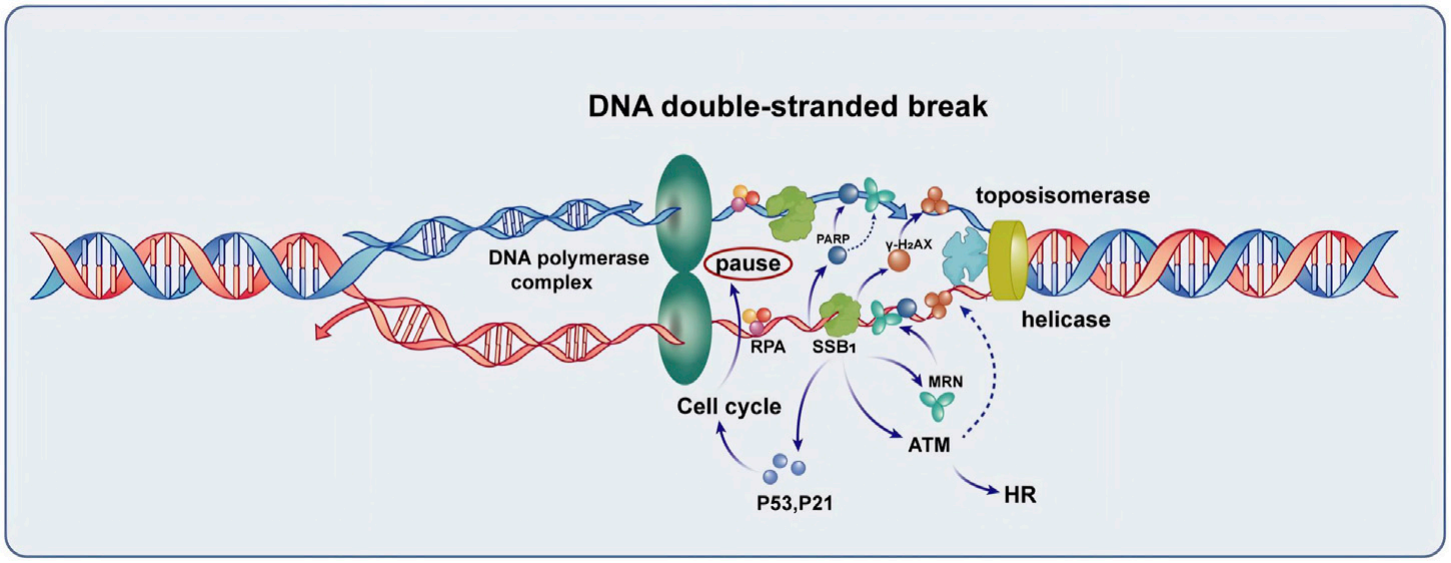
The function of SSB1 in the repair of DNA double-strand break. SSB1 is integral to the repair process, encompassing the protection of single-stranded DNA, recruitment of the MRN complex, coordination with γ-H2AX and PARP repair proteins to enhance signaling for repair, and regulation of cell cycle arrest mediated by P53 and P21. Additionally, SSB1 amplifies ATM kinase activity, thereby accelerating homologous recombination (HR) and overall DNA damage repair processes, making it a crucial protein for maintaining genomic stability.

While the essential role of SSB1 in DNA damage repair has been widely acknowledged (Figure 1), its specific mechanism of action in particular tissues, such as salivary glands, and its dynamic changes in repairing radiation damage still need to be elucidated. This study aimed to investigate the expression pattern of SSB1 in normal rat salivary gland tissues, along with its expression and regulatory changes following radiation exposure, with a focus on its involvement in the repair of salivary gland injury. This study elucidates the importance of examining the role of SSB1 in mitigating radiation damage to salivary glands to understand the biological effects induced by radiation and devise effective strategies for radiation protection and treatment.

## 2 Methods and materials

### 2.1 Experimental animals

A total of 45 adult male Sprague‒Dawley (SD) rats, aged 10 weeks and weighing between 200 and 250 g, were procured from the Experimental Animal Center of Guangxi Medical University. All the rats utilized in this study were maintained in a specific pathogen-free (SPF) environment at a controlled room temperature of 22°C–24°C with a 12/12-h light/dark cycle, allowing free access to food and water. Five rats were randomly assigned to the control group (nonirradiated group, NG), which did not receive radiation therapy. The remaining 40 rats were allocated to the irradiated group (irradiated group, IG) and underwent radiation therapy following an adaptation period. Within 8 h post-radiation treatment, five rats from the experimental group were randomly selected for euthanasia via intraperitoneal injection of an overdose of 3% pentobarbital sodium every h over a total duration of 8 h. Throughout the experiment, all procedures were meticulously conducted in accordance with established ethical principles. No rats were euthanized prior to the conclusion of the experimental protocol, and no rats survived following the completion of the study. Additionally, this experimental protocol received formal approval from the Ethics Committee of Guangxi Medical University (approval number: 202307010) and adhered rigorously to the ARRIVE guidelines to ensure the scientific integrity, ethical compliance, and reproducibility of the study.

### 2.2 Irradiation method

IG rats were anesthetized via intraperitoneal injection of 3% pentobarbital sodium (10 mg/kg) (Solarbio, Beijing) and subsequently secured in position, with lead shields employed to protect surrounding areas. They were irradiated via a 6 MV electron linear accelerator with a radiation field measuring 30 cm × 6 cm at a source–skin distance of 100 cm, receiving a single dose of 6 Gy to the head and neck at a dose rate of 400 cGy/min. Following irradiation, five rats were euthanized every h for 8 h, resulting in eight groups corresponding to different time points. The parotid glands from both the NG and IG rats were harvested and divided into two portions: one portion was fixed in 4% paraformaldehyde solution (Solarbio, Beijing), dehydrated, embedded in paraffin, and reserved for subsequent hematoxylin-eosin staining and immunohistochemical analysis; the other portion was placed in RNA protection solution (TaKaRa Bio-Engineering Co., Ltd.) for long-term storage at −80°C prior to RT-qPCR.

### 2.3 Hematoxylin‒eosin staining

After fixation at room temperature for 24 h, tissue dehydration was performed via an automatic dehydrator (Leica, Japan). Paraffin embedding was carried out via a paraffin embedding machine (Wuhan Kangqiang Medical Devices Co., Ltd.), after which the tissues were cut into 4-micron-thick sections for hematoxylin-eosin staining with paraffin microtome (Thermo Fisher, United States). The stained sections were subsequently examined under an upright fluorescence microscope (Leica, Germany).

### 2.4 Immunohistochemical detection

The prestaining steps were conducted according to the HE staining method. The slides were subsequently incubated at 60°C for 3–4 h, dewaxed with xylene, and subjected to high-temperature EDTA treatment to inhibit endogenous peroxidase activity. The SSB1 monoclonal antibody (Bethyl Laboratories, United States) (1:150) was utilized and incubated overnight at 4°C, followed by treatment with a polymer-assisted horseradish peroxidase conjugate and staining with DAB chromogenic reagent. After dehydration, clarification, and mounting of the samples, five random fields were selected via a 400× upright fluorescence microscope (Leica, Germany), and Image-Pro Plus 6.0 software was used for analysis to determine the average optical density representing the expression level of SSB1.

The specific steps outlined in the experimental protocol were followed, with initial procedures consistent with those previously described. Subsequently, antigen retrieval was performed via citrate buffer, and a γ-H2AX antibody (Bethyl Laboratories, United States, diluted 1:800) along with a PARP1 antibody (Servicebio, China, diluted 1:1200) was applied. The mixture was incubated overnight at 4°C, followed by treatment with a polymer-assisted horseradish peroxidase conjugate and staining with the DAB chromogen. The samples were then dehydrated, cleared, and mounted. Finally, the immunohistochemical images were analyzed via Image-Pro Plus 6.0 software; for each sample at ×400 magnification, five distinct regions were randomly selected to record the number of positive cells. The final count of positive cells was determined by calculating the average across each group.

### 2.5 RT-qPCR detection of SSB1 mRNA expression

In this study, total RNA was extracted from parotid gland samples following the protocol of the Total RNA Extraction Kit (TaKa Ra Takara Biotechnology Co., Ltd.), and its purity and concentration were assessed via a UV‒visible spectrophotometer. The total RNA was then reverse transcribed into cDNA via a reverse transcription kit (TaKaRa Ra Takara Biotechnology Co., Ltd.). The relative expression level of SSB1 gene was subsequently determined via real-time fluorescence quantitative PCR. Details of the primers used can be found in Table 1. The reaction conditions were as follows: (1) Predenaturation: 95°C for 30 s, one cycle. (2) PCR: 95°C for 5 s, 60°C for 30 s, and 40 cycles. To confirm product formation, a portion of the final reaction mixture was subjected to electrophoresis on a 2% agarose gel at 120 V for 40 min after adding nucleic acid dye. Upon completion of the reaction, while the Ct value of each sample was recorded, analysis and confirmation were conducted on the basis of the qPCR amplification curve and melting curve. Next, ΔΔCt = [Ct (SSB1 gene in the radiation group - GAPDH internal reference gene) - Ct (SSB1 gene in the control group - GAPDH internal reference gene)], average ΔΔCt values from three replicate wells of each sample were calculated; subsequently, formulae were utilized to determine the relative expression levels between the irradiated and nonirradiated groups.

**TABLE 1:** Primers for the target gene SSB1 and the reference gene GAPDH.

Gene	Primer sequence	Amplification length
SSB1	Upstream primer 5′-TAT​AAC​ACC​CAG​CAG​GCA​TCC​A-3′	128 bp
	Downstream primer 5′-GTG​CTC​AGT​CCA​TTC​CCG​TTC-3′	
GAPDH	Upstream primer 5′-GGC​ACA​GTC​AAG​GCT​GAG​AAT​C-3′	143 bp
	Downstream primer 5′-ATG​GTG​GTG​AAG​ACG​CCA​GTA-3′	

### 2.6 Statistical analysis

The experimental data are presented as the means ± standard deviations and were analyzed via SPSS 25.0 statistical software. Independent sample *t*-tests were employed to compare the differences between each experimental group and the control group, with statistical significance set at *P* < 0.05. Graphs were created via GraphPad Prism v8.0 software (Graph Pad Inc., San Diego, CA, United States).

## 3 Results

### 3.1 Results of HE staining

**FIGURE 2:**
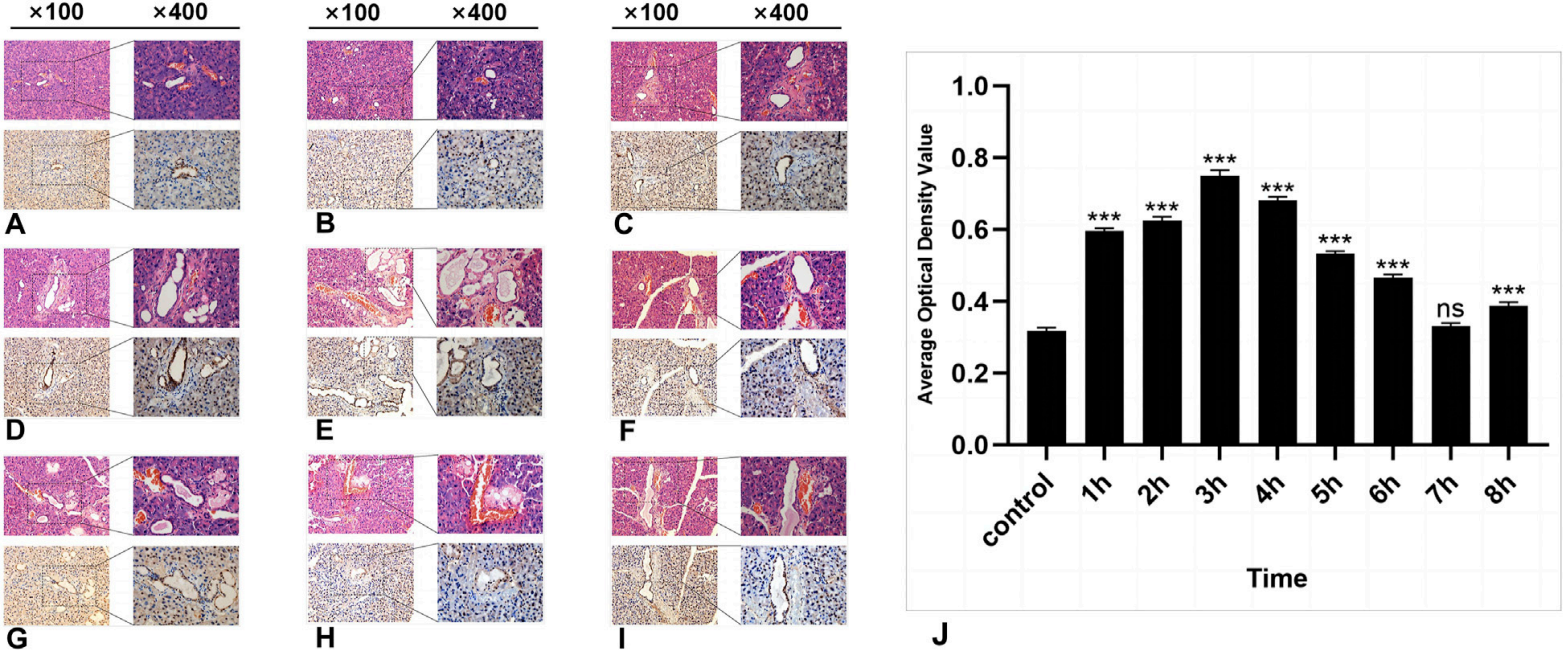
The HE and IHC staining of SSB1 in the parotid gland tissues of the rats. **(A–I)**: Control group and 8 experimental groups, respectively. In the experimental groups, the expression of SSB1 first increased but then decreased over time. The expression was the highest in the 3-h group after radiation **(D)** and then gradually decreased to the normal level. Except for the 7-h group, there were significant differences between the other experimental groups and the control group **(J)**, *P* < 0.05.

HE staining revealed no significant morphological changes in the parotid gland tissue of the experimental group within several h after radiation compared with that of the control group. The ducts, blood vessels, and acinar cells presented clear features. The acinar cells of the parotid gland were closely arranged in a cone-shaped or quasicircular pattern, with nuclei located at their base showing darker staining. The cytoplasm displayed slight eosinophilia, and small vacuoles were visible. Intercalated ducts between the acini were relatively thin in diameter and were lined by a single layer of flat or cuboidal epithelium. Connective tissue septa containing structures such as blood vessels, nerves, and ducts separate lobules and interlobules. The fibrous components in the connective tissue appeared light pink (Figures 2A–J).

### 3.2 Results of immunohistochemical detection of SSB1

**FIGURE 3:**
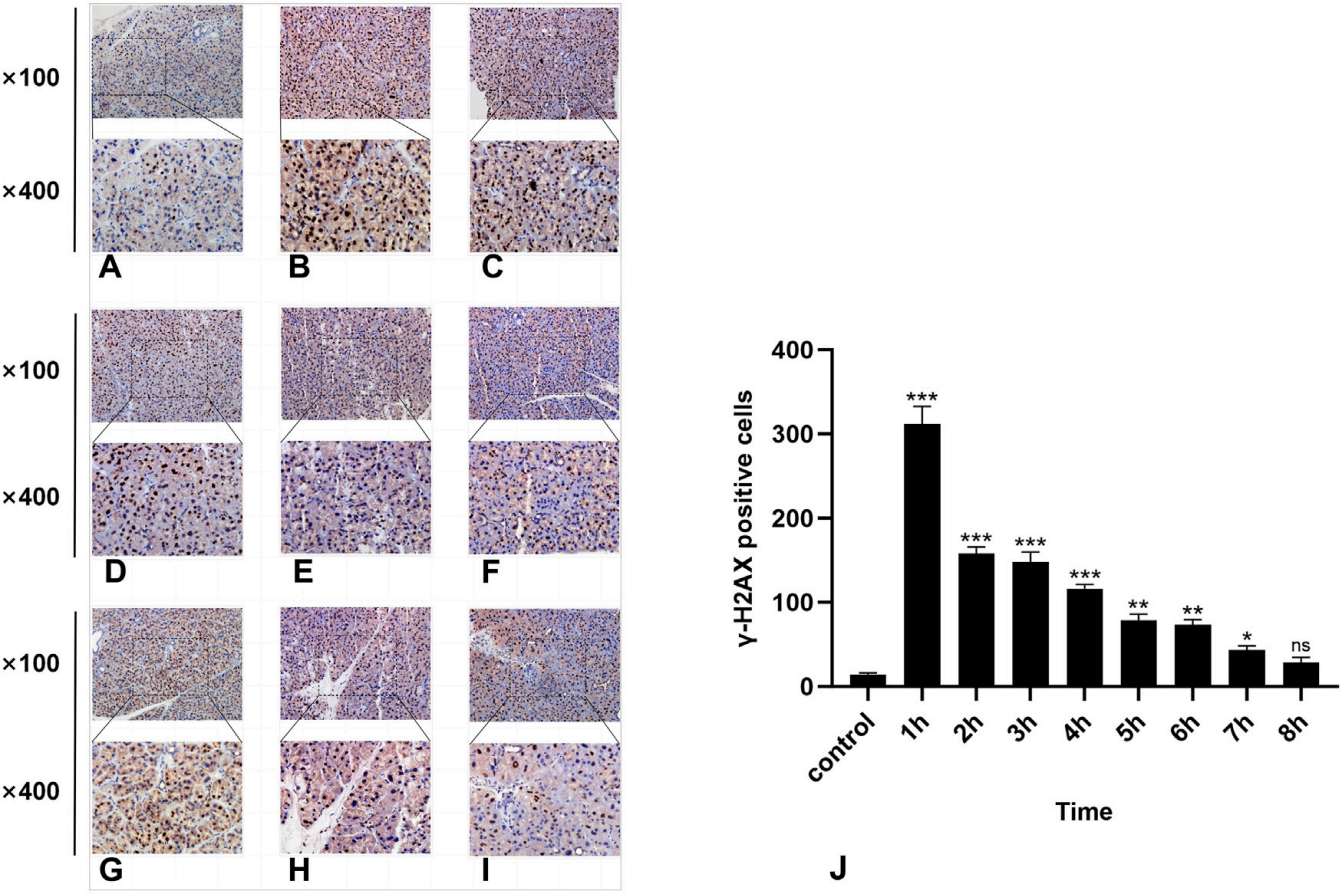
IHC staining of γ-H2AX. **(A-I)** represent the control group and 8 experimental groups, respectively. The positive expression of γ-H2AX was the highest at 1 h **(B)** and then gradually decreased. Except for the 8-h group, there were significant differences between the other experimental groups and the control group **(J)**, *p* < 0.05.

In the control group, a certain level of SSB1 expression was observed in the parotid gland tissue of the mice that did not receive radiation, and SSB1 was primarily localized within the nuclei of acinar cells and ductal cells. Notably, the highest expression level was detected in ductal cells, followed by acinar cells; however, no expression was detected in the connective tissue cells of other parotid gland tissues (Figure 2A). Following radiation exposure, conspicuous SSB1 expression emerges in acinar cells, resulting in an overall pattern of initial elevation followed by reduction. The peak expression occurred at 3 h postradiation and subsequently decreased to near-normal levels (Figures 2A–I; Figure 3J). With the exception of the 7-h time point, statistically significant differences were evident between the experimental group and the control group at all other time points (*P* < 0.05).

### 3.3 Results of immunohistochemical detection of γ-H2AX

In the normal rat parotid gland tissue of the control group, a small number of γ-H2AX-positive cells were observed in the nucleus, exhibiting a brown–yellow color. The radiation group exhibited distinct characteristics at different time points. Notably, the 1-h group presented the highest number of γ-H2AX-positive cells (Figure 3B), while at subsequent time points, there was a significant decrease in the number of γ-H2AX-positive cells over time (Figure 3). Compared with the control group, statistically significant differences were observed at all time points except for the 8-h group (*P* < 0.05).

### 3.4 Results of immunohistochemical detection of PARP1

**FIGURE 4:**
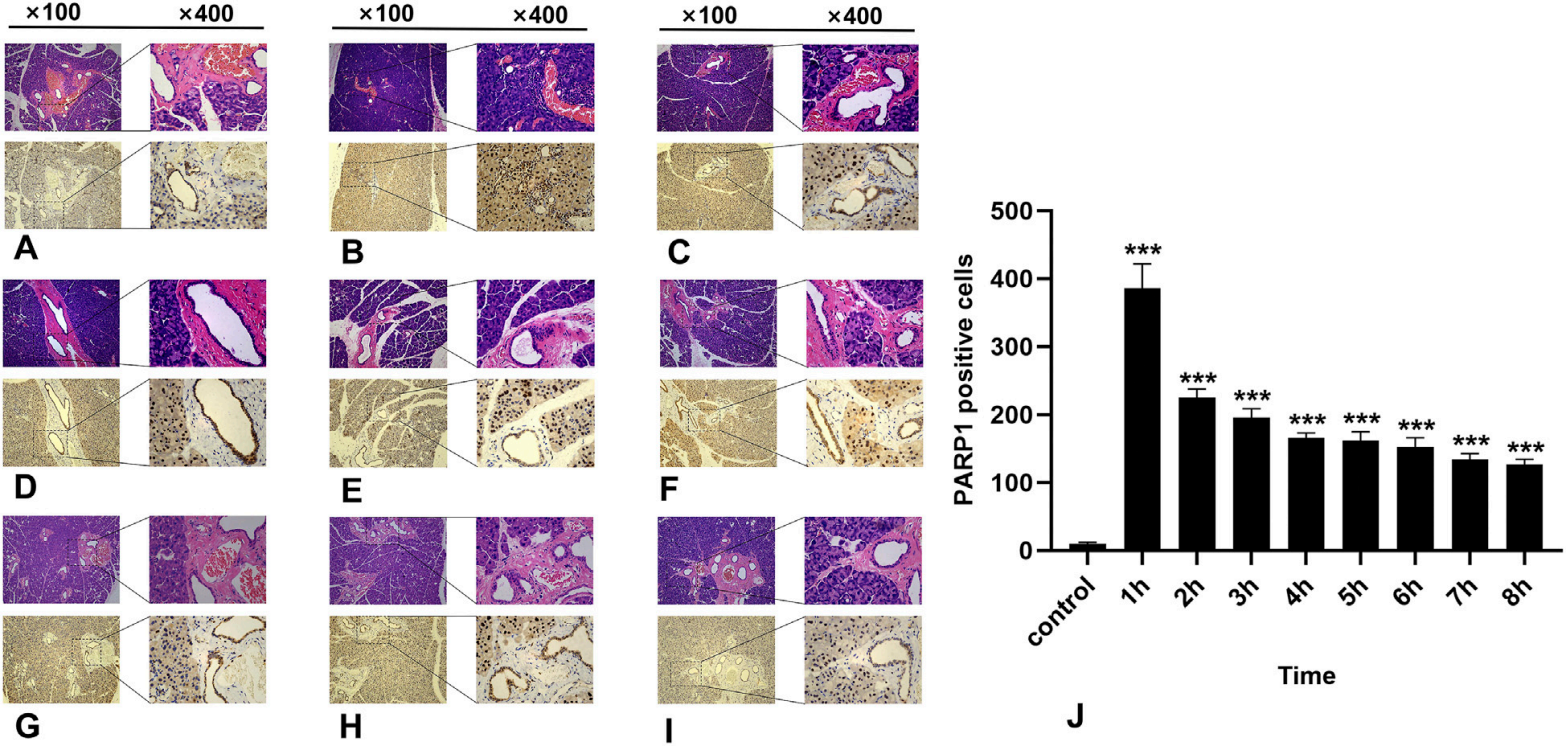
IHC staining of PARP1. **(A-I)** represent the control group and the 8 experimental groups, respectively. The positive expression of PARP1 peaks at 1 h **(B)** and subsequently decreases gradually. Within 8 h following radiation exposure, statistically significant differences were observed between the experimental groups and the control group **(J)**, *P* < 0.05.

In the normal salivary gland tissue of the control group, a limited number of PARP1-positive cells were identified; these cells presented brownish-yellow coloration and were predominantly localized within the cell nuclei. During the first 8 h post-irradiation, PARP1 expression initially increased but then subsequently decreased, with the highest number of PARP1-positive cells recorded in the 1-h group (Figure 4B) (*P* < 0.0001). As time progressed, there was a significant reduction in positive cell counts across all other groups (Figure 4). Notably, significant differences were observed between the experimental and control groups within 8 h following irradiation (*p* < 0.05).

### 3.5 RT-qPCR results

**FIGURE 5:**
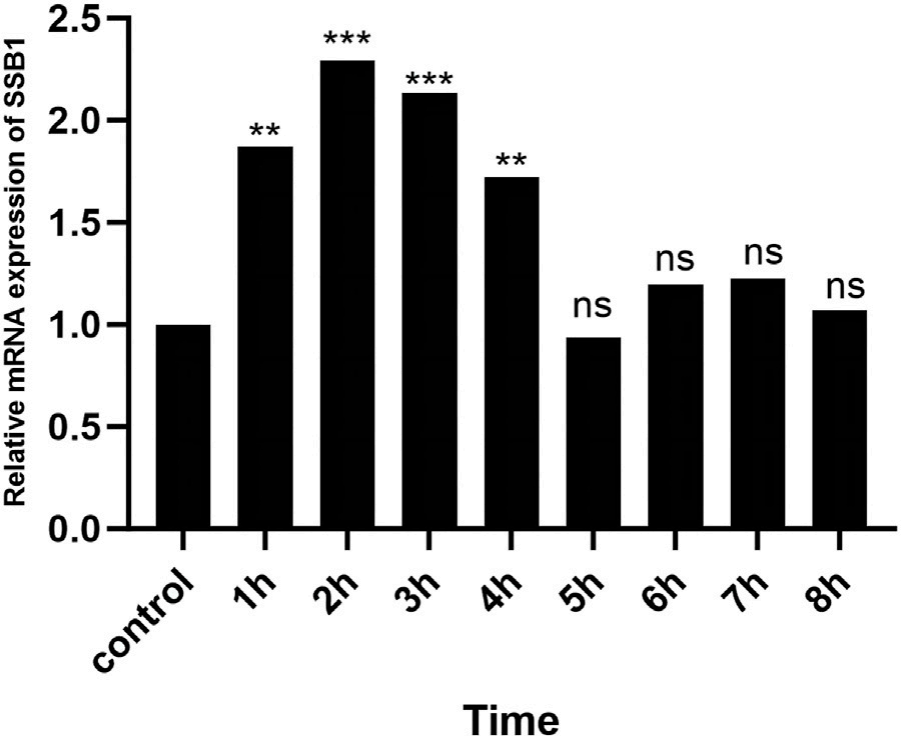
RT-qPCR results of SSB1 mRNA expression. The mRNA expression first increased but then decreased, peaking at 2 h, and there were statistically significant differences compared with that in the control group between 1 and 4 h, *P* < 0.05.

After irradiation, the expression of SSB1 mRNA initially increased but then gradually decreased over time. The peak expression was observed at 2 h, after which it gradually returned to baseline levels by 4 h. Comparative analysis revealed significant upregulation of the target gene SSB1 in the irradiation group at 1–4 h compared with that in the control group (*P* < 0.05) (Figure 5). However, from 5 to 8 h, the expression of SSB1 approached normal levels, with no statistically significant difference (*P* > 0.05).

## 4 Discussion

Current research suggests that the radiosensitivity of cells is partially dependent on the response and repair capacity of DNA double-strand breaks. Additionally, this repair capacity plays a pivotal role in the cellular response following irradiation (Banáth et al., 2010; Taneja et al., 2004; Menegakis et al., 2009; Dunne et al., 2003; Klokov et al., 2006; Liberal and McMahon, 2023). DNA double-strand breaks caused by ionizing radiation represent a predominant and highly consequential type of damage. Failure to promptly initiate repair mechanisms can result in genetic instability and potentially lead to mitotic catastrophe. To preserve DNA integrity, cells have evolved specialized mechanisms for detecting and collaboratively repairing damaged DNA (Ciccia and Elledge, 2010). Upon the formation of DSBs, cells trigger the DNA damage response mechanism and recruit a plethora of proteins associated with DNA repair to the sites of DSBs. In 2008, Richard et al. identified hSSB1 in metazoans, shedding new light on the signal transduction mechanism for DNA damage and revealing its impact on the cellular DNA damage response, which is potentially implicated in tumor prevention and therapeutic responses. Notably, SSB1 is exclusive to complex higher eukaryotes (Richard et al., 2008). Subsequent studies have confirmed that recombinant SSB1 is capable of recognizing single-stranded DNA and plays a crucial role in repairing DSBs. It localizes to sites of DNA double-strand breaks earlier than other proteins involved in DNA repair, such as the MRN complex, and facilitates their recruitment (Richard et al., 2011a; Richard et al., 2011b).

The acute response following radiotherapy encompasses DNA repair, cell cycle arrest, and apoptotic pathways, with the prevalence of specific pathways being tissue-specific. In patients with head and neck cancer undergoing radiotherapy, a common treatment modality for this type of cancer, adjacent normal salivary glands exhibit exceptional sensitivity to therapeutic radiation because of their inability to elicit a robust cell cycle arrest response, consequently leading to cellular apoptosis and reduced saliva secretion (Limesand et al., 2009; Mitchell et al., 2010). Several studies have effectively preserved salivary gland function by employing the cyclin-dependent kinase inhibitor roscovitine to induce the cell cycle arrest response (Martin et al., 2012). In mice subjected to head and neck radiotherapy, there was an increase in DNA double-strand breaks in parotid salivary gland cells following the completion of treatment, with sustained elevation for 3 h. Pretreatment with insulin-like growth factor-1 resulted in the upregulation of SirT-1 protein expression and increased the deacetylation of associated targets, leading to the mitigation of DNA damage and the alleviation of radiotherapy-related symptoms. These findings suggest potential improvements in patients' quality of life (Meyer et al., 2017). Other researchers have verified that Tousled-like kinase 1 (TLK1) is capable of facilitating DNA repair, mitigating radiation toxicity, and preserving the functionality of animal salivary glands (Palaniyandi et al., 2011; Sunavala-Dossabhoy et al., 2012; Timiri Shanmugam et al., 2013). Hence, DNA double-strand breaks play a pivotal role in the cellular damage caused by radiation. Therapeutic approaches aimed at DSBs repair have the potential to mitigate radiation toxicity and preserve salivary gland function during head and neck radiotherapy.

The repair of DSBs involves two primary pathways: nonhomologous end joining (NHEJ) and homology-directed recombination (HR). Both HR and NHEJ are involved in the repair of DSBs induced by ionizing radiation (IR) during the late S/G phase. However, HR specifically operates during the S/G2 phase of replicating cells, utilizing sister chromatids as templates. Cells deficient in either of these pathways exhibit increased sensitivity to IR (Rothkamm et al., 2003; Hinz et al., 2005). SSBs play pivotal roles in the repair of DSBs. First, the concurrent deletion of SSB1 and SSB2 significantly exacerbates HR-mediated DNA repair deficiency, intensifies cellular radiosensitivity, and induces cell apoptosis. Second, in addition to safeguarding single-stranded DNA, SSB can also recruit pertinent cooperative proteins and furnish substrates for them to facilitate diverse DNA damage repair processes. Furthermore, SSB1 can influence various endpoints during DSBs repair, including damage or ablation of Ataxia telangiectasia mutated protein (ATM) mediated damage signal transduction, activation of cell cycle checkpoints, and homologous recombination repair.

Upon the occurrence of DNA double strand breaks, a pivotal event is the phosphorylation of histone H2AX, leading to the formation of the DSBs marker γ-H2AX, which rapidly accumulates at the site of damage to form a “nuclear knot.” The quantity of these structures correlates with the number of DSBs that have occurred (Schmid et al., 2012). Some studies have demonstrated through double-label immunofluorescence that SSB1 can synergize with γ-H2AX to localize at the site of DSBs within 1 min, preceding related proteins such as the MRN complex and persisting for 8 h (Richard et al., 2011a). The MRN complex plays a crucial role as a key protein in homologous recombination repair. The findings of Richard et al. suggest the following relationship between SSB1 and the MRN complex: 1. SSB1 is independently recruited to the site of DSBs whereas MRN facilitates the expansion of SSB1 nuclear nodules. 2. The recruitment of MRN to the break site and the formation of nuclear nodules are dependent on SSB1; 3. SSB1 directly interacts with NBS1, forming a novel functional complex with MRN that enhances its endonuclease activity (Richard et al., 2011a; Richard et al., 2011b). Moreover, in another study, we reported that silencing the SSB1 gene led to a reduction in NBS1 expression in the rat submandibular gland. Furthermore, IR impacts the regulation of both SSB1 and NBS1, resulting in a synergistic effect on the inhibition of NBS1 and the repair of DSBs induced by IR (Gao et al., 2024). SSB1 clearly closely associates with the MRN complex and collaboratively participates in the process of homologous recombination repair.

The cytokines P53 and P21 are pivotal in orchestrating the G1/S transition and the G2/M checkpoint during cell cycle regulation. Upon the occurrence of DSBs, activation of the DNA damage checkpoint by P53 and P21 halts cell cycle progression, allowing time for repair. Xu et al. initially demonstrated that SSB1 directly interacts with P53 and P21 to inhibit their ubiquitination and degradation (Xu et al., 2011). Furthermore, hSSB1 is capable of modulating the activities of P53 and P21 at both the transcriptional and posttranscriptional levels, thereby ensuring their stability and facilitating the proper functioning of the G1/S transition and G2/M checkpoints. Recent investigations have revealed that hSSB1 negatively regulates p53 and RNA polymerase II transcription during radiotherapy for prostate cancer, thus playing a pivotal role in mediating cellular responses to androgen signaling and DNA damage (Adams et al., 2023). Furthermore, research conducted by Xu et al. suggested that SSB1 plays a critical role in maintaining chromosomal stability and preventing genomic instability. Deletion of SSB1 may result in impairment of the cell DNA damage checkpoint and disruption of the cell cycle process (Xu et al., 2011; Xu et al., 2013; Xu et al., 2023).

The ATM kinase-mediated signal transduction pathway represents a critical mechanism for cellular survival following radiation exposure. Research indicates that inhibition of ATM kinase activity leads to destabilization of SSB1, whereas suppression of SSB1 activity restricts the phosphorylation of ATM kinase and its downstream target proteins (Richard et al., 2008). This study also confirmed that SSB1 is a substrate of ATM kinase and can undergo phosphorylation. Phosphorylated SSB1 significantly augments the activity of ATM kinase, thereby bolstering signal transduction and expediting the entire repair process. It is evident that SSB1 and ATM kinases directly interact with each other, exerting mutual influence and actively participating in the process of DNA damage signal transduction.

In this study, we employed immunohistochemistry and RT-qPCR techniques to comprehensively investigate the expression of SSB1 in rat parotid glands at both the protein and gene levels. Our findings provide the first insight into the basal expression pattern of SSB1 in normal rat salivary gland tissues. We observed a nonuniform distribution of SSB1 expression in parotid gland tissue, indicating a certain degree of tissue specificity. The immunohistochemistry results revealed endogenous SSB1 expression in unirradiated rat parotid gland tissue from the NG group, which was predominantly localized within the nuclei of acinar and ductal cells. Significantly different levels of SSB1 content were detected at various locations, with the highest abundance in ducts followed by acini, while minimal to no expression was observed in connective tissue. Previous studies have consistently demonstrated that serous cells are more susceptible to radiation than ductal cells in salivary gland tissues. Considering the functional characteristics of SSB1, we hypothesize that the phenomenon observed in this study may be attributed to the diverse roles of SSB1 in different cell types within the salivary gland, potentially linked to their respective cellular functions and DNA metabolic activities. The literature suggests that SSB1 is involved not only in cell replication processes but also in safeguarding telomere ends (Bolderson et al., 2014; Gu et al., 2013; Pandita et al., 2015). Subsequent studies have also demonstrated that mouse embryos lacking the SSB1 allele present phenotypes such as impaired growth and development, skeletal abnormalities, and intestinal atrophy characterized by diminished stem cells and progenitor cells, ultimately leading to perinatal mortality. Furthermore, adult mice deficient in the SSB1 gene display increased susceptibility to cancer, compromised male reproductive function, heightened sensitivity to ionizing radiation, and reduced genetic stability (Shi et al., 2013; Feldhahn et al., 2012; Shi et al., 2017). It is evident that SSB1 not only plays a role in the repair of DSBs but also contributes to various other cellular metabolic activities.

In a quiescent cellular state, SSB1 is unstable. Upon phosphorylation triggered by factors such as radiation and cytotoxic damage, its stability is increased, leading to upregulated expression that can persist for more than 8 h (Richard et al., 2011a; Richard et al., 2008). The results of this experiment also yielded a similar conclusion. Eight h after radiation treatment, the expression of SSB1 in rat salivary gland tissue significantly changed in a time-dependent manner, with the expression level of SSB1 initially increasing but then subsequently decreasing. During the early stages of radiation exposure (0–2 h), there was a rapid increase in the expression level of SSB1, which was closely associated with increased DNA damage and single-stranded DNA exposure induced by radiation. As an early response protein, SSB1 rapidly associates with these exposed single-stranded DNA, thereby safeguarding them from degradation and providing a platform for subsequent repair processes. Following this (2–4 h), upon the initiation of the DNA repair mechanism and the completion of single-stranded DNA repair, the expression of SSB1 begins to decrease. Ultimately (4–8 h), the expression level of SSB1 gradually reverted to a near-normal state, indicating effective DNA damage repair and entry into a relatively stable cellular state. The emergence of this trend is in line with the kinetics of DSBs repair, indicating that the upregulation of SSB1 expression may be partially involved in DSBs damage repair. On the basis of these findings, SSB1 may play a pivotal role in the repair process of radiation-induced damage to salivary glands. First, the swift upregulation of SSB1 serves as an early cellular response mechanism to radiation damage, aiding in stabilizing single-stranded DNA and preventing its further degradation. Second, SSB1 can recruit other repair proteins to the site of damage and facilitate prompt DNA damage repair. Finally, upon completion of the repair process, the expression level of SSB1 gradually reverts to normal, reflecting an effective cellular response and recovery from damage.

Furthermore, immunohistochemistry analysis revealed that the highest number of γ-H2AX-positive cells was observed at 1 h, followed by a significant decrease toward normal levels in the later stages. This observation aligns with previous research findings and may be attributed to DSBs repair, dephosphorylation of γ-H2AX to H2AX, or clearance of γ-H2AX due to cellular apoptosis. Owing to the 1-h set time interval, it is not possible to definitively determine that 1 h represents the peak value. Intriguingly, we also observed γ-H2AX-positive cells in normal parotid gland tissues. Previous studies have indicated that certain DSBs may arise from the endogenous metabolic processes of the cell; thus, our findings are not contradictory.

Furthermore, SSB1 is more persistent than γ-H2AX in radiation-induced DSBs and forms “nuclear nodules” in cooperation with γ-H2AX at the DSBs site (Richard et al., 2011a; Limesand et al., 2006). This experiment yielded similar findings. A comparison of the expression patterns of SSB1 and γ-H2AX in the parotid glands of rats at 8 h revealed a congruent overall trend, albeit with a more rapid decline in γ-H2AX than in SSB1. These findings suggest that the alteration in SSB1 expression at this time point parallels the DNA damage repair process, suggesting that the upregulated fraction of SSB1 may be involved in DSBs damage repair. Within a specific range of sublethal radiation doses, cells possess the capacity for self-repair. Following the repair of damage, cells downregulate the activity or expression of relevant repair proteins through a series of protein modification mechanisms, such as dephosphorylation and deacetylation. Owing to the distinct status and functions of various repair proteins in DSBs repair, their respective action times also vary. Celeste et al. reported that in the absence of H2AX, the recruitment of repair proteins to sites of double-strand breaks is still possible; however, their mobility is reduced, and sustained aggregation becomes challenging (Paull et al., 2000). These findings suggest that γ-H2AX may specifically facilitate the aggregation and maintenance of associated repair proteins at the sites of DSBs without directly engaging in DSBs signal transduction and repair.

Consequently, following radiation exposure, γ-H2AX expression rapidly and substantially increases, reaching a peak before subsequently declining significantly. In contrast, SSB1 exerts a pervasive influence across all stages of the DNA damage response and can persist for more than 8 h (Richard et al., 2011a; Richard et al., 2008). Hence, a certain level of SSB1 can still be detected during the advanced stages of radiation therapy.

The poly (ADP‒ribose) polymerase (PARP) family plays a pivotal role in cellular physiology and pathology, encompassing critical processes such as DNA transcription regulation, maintenance of genome stability (including the DNA damage response and repair), cell cycle regulation, and apoptosis. PARP1 and PARP2 serve as early-recognition molecules for DSBs, which are essential for the initiation of DSBs repair mechanisms. They rapidly recruit subsequent repair factors, including the MRN complex (Haince et al., 2008). Notably, PARP1—the most abundant and functionally versatile member of this family—is a key enzyme involved in single-strand break repair, facilitating single-strand break recognition and repair through the base excision repair (BER) pathway (Michels et al., 2014). Inhibition of PARP1 results in the conversion of single-strand breaks into more severe double-strand breaks during replication, establishing a theoretical foundation for its role as a target in cancer therapy (McGlynn and Lloyd, 2002). Currently, PARP inhibitors have demonstrated significant potential in treating breast cancer, ovarian cancer, pancreatic cancer, and prostate cancer, particularly when combined with radiation therapy; this combination markedly enhances therapeutic efficacy by promoting DSBs accumulation within tumors (Benafif and Hall, 2015). PARP1 utilizes nicotinamide adenine dinucleotide (NAD+) as a substrate and rapidly associates with the site damaged by single-strand breaks to activate polymerase activity and recruit interacting proteins such as ALC1/CHD1L and XRCC1 to facilitate the repair process (Tsuda et al., 2017; El-Khamisy et al., 2003; Satoh and Lindahl, 1992). Experimental evidence indicates that following radiation-induced high levels of DNA damage in rat germ cells, integrity can be restored *in vitro* within 1–3 h; however, this recovery process is prolonged by the addition of PARP inhibitors, underscoring the essential role of PARP ineffective repair (Atorino et al., 2001).

Similarly, investigations of CHO-K1 cells revealed a significant increase in DSBs within 3 h of exposure to multiple PARP inhibitors, further corroborating the central function of PARP in the DNA damage response (Boulton et al., 1999). Furthermore, this study revealed that the expression of PARP1 in the salivary gland tissue of rats increased rapidly after radiation exposure and reached its peak within 1 h, remaining at a high level for up to 8 h. Compared with that of γ-H2AX, its duration is extended, and its expression trend aligns more closely with that of SSB1, suggesting the potential involvement of SSB1 in repair processes following radiation injury to salivary gland tissue. This dynamic change not only reflects the rapid responsiveness of the tissue to radiation stimuli but also highlights the need for further exploration into how other related proteins collaborate to achieve comprehensive and effective DNA repair.

While this study highlights the crucial role of SSB1 in repairing radiation-induced injury to salivary glands, further exploration is needed to elucidate its specific mechanism. In our subsequent research, we will employ techniques such as gene knockout, Western blotting, and coimmunoprecipitation to elucidate further the involvement of SSB1 in repairing radiation-induced injury to salivary glands and enhance the understanding of the underlying mechanisms governing radiation sensitivity in these glands. Future research should focus on the following aspects: first, conducting an in-depth investigation into the interaction between SSB1 and other repair proteins (such as DNA polymerase and ligase) and their synergistic repair mechanisms; second, exploring the role of SSB1 in stress responses such as radiation-induced apoptosis and autophagy; and third, examining the differences in SSB1 expression changes and their biological significance under various radiation doses and irradiation conditions. Furthermore, these findings can also have clinical implications for exploring the potential of SSB1 as a biomarker and therapeutic target for radiation injury.

## 5 Conclusion

This study demonstrated that SSB1 in rat salivary gland tissue plays a crucial role in the repair of radiation-induced injury. Furthermore, radiation can induce the upregulation of SSB1 expression and its involvement in the DNA damage repair process. These findings not only increase our understanding of the mechanism of SSB1 in DNA damage repair but also establish an essential theoretical foundation and experimental reference for the future development of effective therapeutic strategies for radiation-induced injury to the salivary glands.

## Data Availability

The raw data supporting the conclusions of this article will be made available by the authors, upon reasonable request.
